# Knowledge, attitudes, and associated factors of cervical cancer screening among women in Debre Markos town, Northwest Ethiopia: a cross-sectional study

**DOI:** 10.1038/s41598-025-18296-0

**Published:** 2025-10-06

**Authors:** Bewket Yeserah Aynalem, Kiber Temesgen Anteneh, Mihretu Molla Enyew

**Affiliations:** 1 Department of Midwifery, Debre Markos University, Debre Markos, Ethiopia; 2 Department of Midwifery, University of Gondar, Gondar, Ethiopia

**Keywords:** Cervical cancer, Screening, Knowledge, Attitude, Debre markos, Ethiopia, Health care, Medical research

## Abstract

Cervical cancer is the leading cause of cancer-related mortality among young women globally, resulting in a significant number of deaths each year. Despite the well-established benefits of cervical cancer screening, its uptake is often influenced by women’s knowledge and attitudes toward the screening process. Considering this, the present study was conducted to evaluate the level of knowledge about cervical cancer, the attitudes toward screening, and the factors associated with these outcomes among women in Debre Markos Town, Northwest Ethiopia. This study was designed as a community-based cross-sectional survey, focusing on women aged 30 to 49 years living in Debre Markos Town. A multistage sampling technique was used to select a total of 630 participants for the study, which was conducted between July 1 and August 30, 2018. Data was entered using EPI Info version 7, while cleaning and analysis were done with SPSS version 25. Initially, bivariable logistic regression was applied to assess the relationships between the outcome variables and potential factors. Then, multivariable logistic regression analysis was employed to identify the independent factors associated with each outcome. A p-value of less than 0.05 was considered statistically significant. The study found that 374 (59.4%) of the participants had adequate knowledge about cervical cancer, while 385 (61.1%) displayed favorable attitudes toward cervical cancer screening. Key factors associated with a knowledge of cervical cancer screening included a history of multiple sexual partners [AOR: 1.768 (95% CI: 1.227, 2.549)] and the use of modern family planning methods [AOR: 2.238 (95% CI: 1.410, 3.554)]. In terms of attitudes toward screening, significant factors included higher educational attainment (college education or above) [AOR: 2.006, 95% CI: 1.147, 3.508], single, divorced, or widowed [AOR: 2.101 (95% CI: 1.219, 3.620)], and more than five pregnancies [AOR: 1.830 (95% CI: 1.125, 2.976)]. The results of this study indicate that both knowledge and attitudes toward cervical cancer screening were below optimal levels among the women in Debre Markos.

## Introduction

Cervical cancer^1^ is an oncogenic neoplasia arising from the cervix, which is the lower segment of the uterus. CC occurs when cells that form the covering of the cervix begin to demonstrate abnormal growth and, as such, can migrate to other parts of the body. About 70% of cervical cancer has been associated with the Human Papillomavirus (HPV)^2,3^. Screening for cervical cancer is the use of a sequence of tests of various kinds among symptom-free subjects to find people who may turn out to be at risk or in an initial stage of disease development^4,5^. Cervical cancer is among the most significant universal public health conditions and one of its major causes of long-term non-communicable disease^5^.

Global reports in 2016 indicate 527,600 cervical cancer incidence cases, 90% of which occur in low- to middle-income countries (LMICs), particularly in sub-Saharan Africa (SSA), where cervical cancer remains at the forefront of female deaths^6^. Cervical cancer kills almost without any symptoms in women, especially in the early stages^7^. Delayed diagnosis occurs more often with possible clinical presentations like vaginal bleeding, invasion, metastasis, and poor prognosis^8^. Cervical carcinoma causes a huge economic burden: direct health system costs, community and household costs, and opportunity costs of lost productivity resulting from premature death and disability^9^. The epidemiological picture of cervical cancer is blessedly rare, other than these fatalities in advanced economies due to adequate screening for preinvasive disease^10^.

Mortality from cervical cancer is still extremely high in resource-poor countries^11^. In Africa, cervical cancer continues to be a factor in morbidity and mortality among women^12^. In Ethiopia, there are approximately 7095 new cases of cervical cancer and 4732 deaths annually^13^. The silver lining is that cervical cancers are nearly entirely preventable. As the disease has a long pre-invasive stage, precancerous lesions may be detected and treated to prevent them from developing into invasive diseases^14^. Cervical cancer screening is recommended by the WHO in women aged 30–50 years, as young women will spontaneously resolve minor lesions^3^. HIV-infected women should also undergo regular screening, as they are more susceptible to infection with HPV^1^.

The WHO Information Center reports high heterogeneity in the extent of cervical cancer screening coverage among different countries^15^. The accessibility and availability of health services are typically accompanied by good knowledge and a positive attitude toward cervical cancer and screening^16^. WHO recommended cervical cancer screening for women between the ages of 30–49 years, and the Ethiopian Ministry of Health accepted the WHO-recommended cervical cancer screening^17,18^.

This nationwide support demonstrates how the government acknowledges the importance of appropriate screening in reducing the disease’s effects. However, merely having policies and recommendations does not guarantee that they will be implemented successfully^19^. A woman’s decision to use essential cervical cancer screening services is greatly influenced by her attitude and level of knowledge about the screening procedure^20^. Even the most well-designed programs will struggle to reach their full potential if there is a lack of awareness and a positive attitude toward screening. Therefore, this study aims to investigate knowledge about cervical cancer, attitudes towards screening, and determinants that affect these attitudes among women living in Debre Markos Town, Northwest Ethiopia.

## Methods

All methods were carried out according to relevant guidelines and regulations.

### Study setting and period

The study setting was Debre Markos, Amhara region, Northwest Ethiopia, from July 1 to August 30, 2018. Debre Markos town is the capital of East Gojjam Zone of Amhara regional state, Northwest Ethiopia, located 300 km away from Addis Ababa, the capital city of Ethiopia, and 265 km from Bahir Dar, the capital city of Amhara region. There are six government health institutions and five non-governmental clinics that provide different reproductive health services in the town. However, there is only one health institution, “Debre Markos Hospital,” that has a cervical cancer screening center.

### Study design

A community-based cross-sectional study design was used.

### Study participants

The source population was all women in the age group 30–49 years in Debre Markos town. The study population was all women in the age group 30–49 years living in Debre Markos town during the study period in the selected kebeles. Those who lived for less than 6 months in Debre Markos town and those who were very ill during data collection were excluded from the study.

### Sample size

The sample size was calculated based on the assumption of the formula for a single population proportion. The proportion of good knowledge and positive attitude towards cervical cancer screening was 53.7% and 65.2% respectively, from the previous study in Ethiopia at Hosanna town^21^, and a margin of error of 5% was taken into consideration.

$$\:\text{I}\text{n}\text{i}\text{t}\text{i}\text{a}\text{l}\text{s}\:\text{a}\text{m}\text{p}\text{l}\text{e}\:\text{s}\text{i}\text{z}\text{e}\:\text{f}\text{o}\text{r}\:\text{k}\text{n}\text{o}\text{w}\text{l}\text{e}\text{d}\text{g}\text{e}={\left(\text{Z}\frac{\text{a}}{2}\right)}^{2}\text{*}\frac{\text{p}(1-\text{p})}{\text{w}2}={1.96}^{2}\text{*}\frac{0.537\left(1-0.537\right)}{({0.005)}^{2}}=382$$

Since it had two stages, we have 1.5 design effects for both outcome variables.

Then this is calculated as 382*1.5 = 573.

We have added a non-response rate of 10% and 573*0.10 = 57.

Then the final sample size was 573 + 57 = 630.

The sample size for attitude was also calculated similarly.

$$\:\text{i}\text{n}\text{i}\text{t}\text{i}\text{a}\text{l}\:\text{s}\text{a}\text{m}\text{p}\text{l}\text{e}\:\text{s}\text{i}\text{z}\text{e}\:\text{f}\text{o}\text{r}\:\text{a}\text{t}\text{t}\text{i}\text{t}\text{u}\text{d}\text{e}={\left(\text{Z}\frac{\text{a}}{2}\right)}^{2}\text{*}\frac{\text{p}(1-\text{p})}{\text{w}2}={1.96}^{2}\text{*}\frac{0.652\left(1-0.652\right)}{({0.005)}^{2}}=349$$

We have used 1.5 design effects, and it has been calculated as 349*1.5 = 524.

Again, a 10% non-response rate was added and calculated as 524*0.10 = 52. Then the final sample size was 524 + 52 = 576. We took the larger sample size, which was 630.

### Sampling techniques

**Fig. 1:**
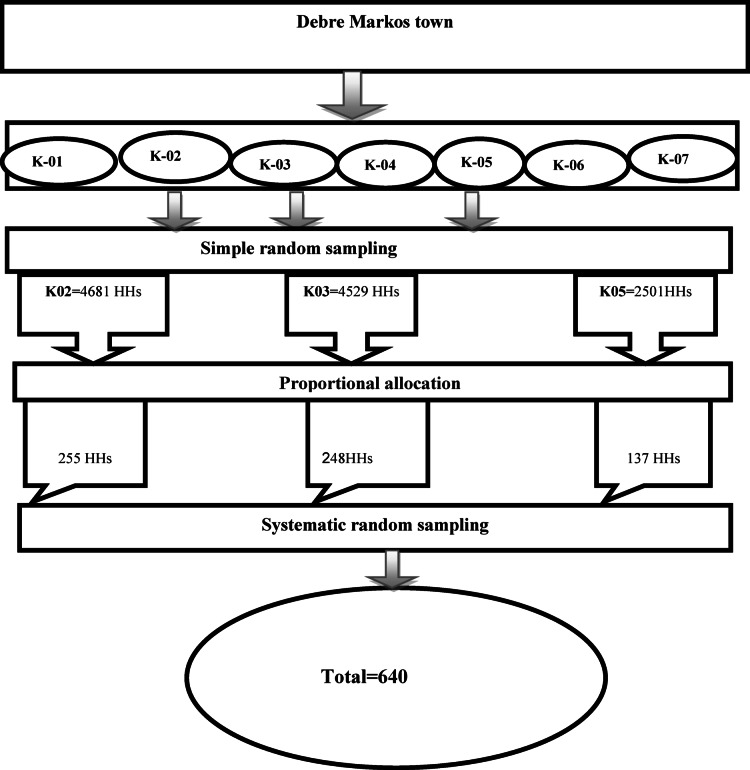
Schematic presentation of sampling procedure to assess Knowledge, Attitudes, and associated factors of cervical cancer screening among women (*n* = 630) in Debre Markos town, Northwest Ethiopia, 2018. K = Kebele; HHs = Households.

A multistage sampling technique was employed using the lottery method, simple random sampling to select 3 Kebeles (the smallest administrative unit in Ethiopia) from 7 Kebeles in Debre Markos town, and get the list of available and coded households in each Kebele (Fig. 1). The number of households consisting of eligible populations to be selected from each kebele was determined proportionally to the size of study units, and the kth value was computed for each selected kebele. The women of the selected household were interviewed, and in case of the presence of more than one woman in the household, the lottery method was used to select only one. In the event of absenteeism, the next available woman was recruited to the study after three revisit attempts.

### Study variables

#### Dependent variables

Knowledge and attitude towards cervical cancer screening.

#### Independent variables

Socio-demographic characteristics, Reproductive and behavioral characteristics.

### Operational definitions

#### Knowledgeable

study participants who scored the knowledge questions a mean or above were regarded as knowledgeable.

#### Positive attitude

study participants who scored the attitude questions a mean and above were regarded as having a positive attitude.

#### Multiple sexual partners

study participants who had more than one penetrative sexual partner in their life history^22^.

#### Cigarette smoking

study participants who had at least one history of smoking in their life, excluding passive smokers^23^.

### Data collection and data quality control

The interviewer administered a structured and pre-tested questionnaire implemented by three trained diploma Midwives and three BSc midwives’ supervisors. Data collectors and supervisors were given two days of training on data collection. The questionnaire was originally prepared in English and translated into the local language, and then back-translated to English. A pretest was done by Direct Questioning on 32 women of the sample size in Dembecha town, and the necessary correction was undertaken whenever it was required.

### Data processing and analysis

The data collected were entered into EPI Info version 7 and exported to SPSS version 25 for data cleaning and analysis. Descriptive statistics were computed to describe the study population using relevant variables. Binary logistic regression was used in identifying determinants of the outcome variable. Independent variables with a P-value < 0.25 upon bivariate logistic regression were subjected to multivariable logistic regression analysis. Variables with a P-value < 0.05 at a 95% confidence level were finally reported as significantly associated with the outcome variable. The strength & direction of the association were interpreted based on the adjusted odds ratio.

### Ethics approval and consent to participate

The ethical feasibility of the study was reviewed and cleared by the Ethical Review Committee of the Department of Midwifery, by delegation of the University of Gondar’s institutional review board (IRB) before data collection. Ethical clearance and support letters were submitted to Debre Markos’s health office, and approval was obtained. A written informed consent was obtained from all participants. Finally, the women were interviewed in private rooms alone at home, the data were anonymous, and the participants had the right to withdraw from the study without any restriction.

## Results

### Socio-demographic characteristics of the respondents

All 630 women selected for the study participated, providing a 100% response rate. The study participants were predominantly women aged 30–39 years (80.8%), which aligns with the WHO-recommended screening age group. A large majority were married (80%), suggesting stable partnership contexts that may influence reproductive behaviors. Nearly all participants identified as Christian (95.4%) and Amhara in ethnicity (98.3%), reflecting the homogeneity of the local population.

Educational attainment was relatively low: 27.9% had no formal education, which may have implications for health literacy and screening uptake. In terms of occupation, the largest group was self-employed (35.4%), followed by government employees (23.2%) and private employees **(**22.5%). Household monthly income varied, with 37.9% earning 1601–2699 birr, indicating a predominance of low- to middle-income households (Table 1).

**Table 1:** Sociodemographic characteristics of women (*n* = 630) aged 30–49 in Debre Markos town, Northwest ethiopia, 2018.

Variable	Frequency	Percent
Age of mothers		
30–39	509	80.8
40–49	121	19.2
Marital status		
Married	504	80
Others*	126	20
Religion		
Christian	601	95.4
Muslim and protestant	29	4.6
Educational status		
No formal education	176	27.9
Primary education	149	23.7
Secondary education	168	26.7
College and above	137	21.7
Ethnicity		
Amhara	619	98.3
Others**	11	1.7
Occupation		
Housewife	119	18.9
Self-employee	223	35.4
Private employee	142	22.5
Government employee	146	23.2
Household income ^***^		
<900	131	20.8
900–1600	129	20.5
1601–2699	239	37.9
>=2700	131	20.8

* Single, divorced, and widowed, ** Oromo and Gurage, ***in Ethiopian Birr.

### Reproductive and behavioral characteristics

The reproductive history of the participants provides important context for understanding cervical cancer risk factors. A substantial majority of the women (78.1%) reported initiating sexual activity after the age of 16, although a noTable 21.9% began at age 16 or younger, potentially increasing their lifetime exposure to HPV. About 40.3% of the women had a history of multiple sexual partners (MSP), which is a known risk factor for cervical cancer. The use of modern family planning (FP) methods was common among respondents (83.8%), with 80.6% using them for 1 to 3 years. Additionally, 83.3% of participants were pregnant at least once, and 80.4% had given birth. Although only 11.1% reported a family history of cervical cancer, 14% had experienced sexually transmissible diseases (STDs) (Table 2).

**Table 2:** Reproductive characteristics of women (*n* = 630) aged 30–49 in Debre Markos town, Northwest ethiopia, 2018.

Variable	Frequency	Percent
Age at which sexual intercourse (in years)		
<=16	138	21.9
>16	492	78.1
Multiple sexual partners		
No	376	59.7
Yes	254	40.3
History of smoking		
No	617	97.9
Yes	13	2.1
History of STD		
No	542	86
Yes	88	14
Ever use a modern FP method		
No	102	16.2
Yes	528	83.8
Duration of modern FP method use		
1–3 years	425	80.6
>=4 years	103	19.4
Family history of cervical cancer		
No	560	88.9
Yes	70	11.1
Ever gotten pregnant		
No	105	16.7
Yes	525	83.3
Gravidity		
1–5	408	77.3
>5	117	22.7
Ever given birth		
No	123	19.6
Yes	507	80.4
Parity		
1–5	418	82.4
>5	89	17.6

### Knowledge and attitude toward cervical cancer screening

**Fig. 2:**
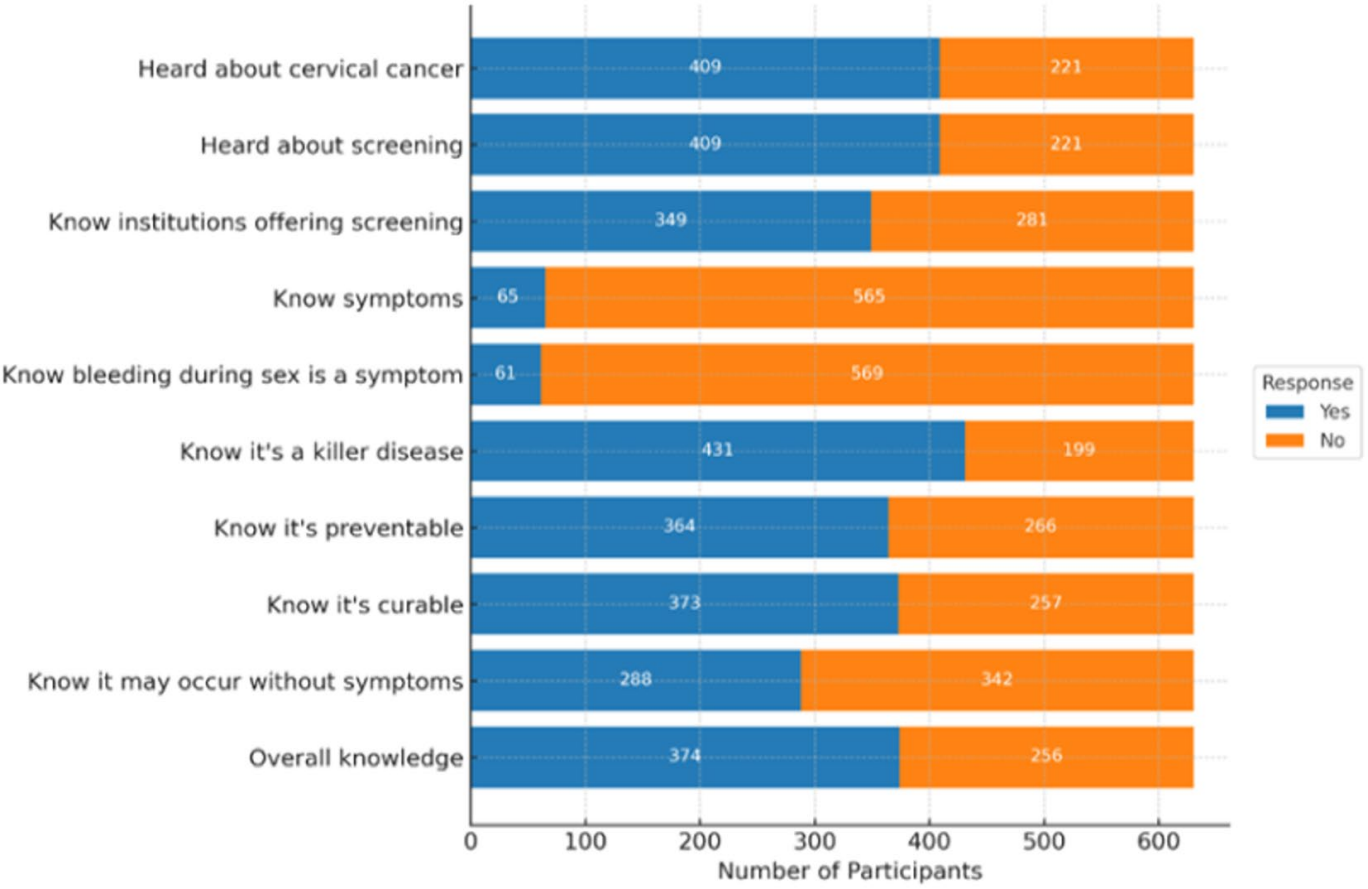
Showing Knowledge and Awareness of cervical cancer screening among women (*n* = 630) in Debre Markos town, Northwest Ethiopia, 2018.

About 374 (59.4%) of the participants knew about cervical cancer screening [95% CI: 55.5, 63]. Most women (64.9%) had heard about cervical cancer and cervical cancer screening. However, only 55.4% knew a health facility that provides screening services. Knowledge of specific symptoms was very low: only 10.3% could mention any symptoms, and 9.7% identified bleeding during sexual intercourse as a warning sign. While 68.4% of participants believed cervical cancer is a fatal disease, fewer understood its preventability (57.8%) and curability (59.2%). Nearly half (45.7%) were aware that cervical cancer can occur without visible signs or symptoms (Fig. 2).

Three hundred eighty-five (61.1%) had a favorable attitude towards cervical cancer screening [95% CI: 57.4, 65]. Nearly half of the respondents (47.5%) agreed that other women are susceptible to cervical cancer, and the same proportion acknowledged their vulnerability to the disease. However, about a quarter remained indifferent or unsure, which suggests uncertainty or a lack of personal relevance attached to the condition. Awareness about the hereditary nature of cervical cancer was particularly low: only 14.8% believed that it could be genetically transmitted, while the majority disagreed or were uncertain. On a more encouraging note, over 60% of women recognized the serious consequences of cervical cancer and acknowledged the benefits of early screening. Despite this, perceptions of the screening procedure itself were divided 57% viewed it as problematic, indicating possible fear, cultural concerns, or misinformation (Table 3).

**Table 3:** Attitude towards cervical cancer and its screening among women (*n* = 630) in Debre Markos town, Northwest ethiopia, 2018.

Variables	Level of agreement					
	Agree		Disagree		Indifferent	
	Number	Percent	Number	Percent	Number	Percent
Susceptibility of other women to cervical cancer	320	47.5	148	23.5	162	25.7
Self-susceptibility to cervical cancer	299	47.5	170	27	161	25.6
Genetic transmission of cervical cancer	93	14.8	354	56.2	183	29
The bad outcome of cervical cancer	383	60.8	133	21.1	114	18.1
Benefits of cervical cancer screening	387	61.4	112	17.8	131	20.8
The problem with the screening procedure	359	57	109	17.3	162	25.7
**Overall attitude**	**Frequency**			**Percent**		
Favorable attitude	385			61.1		
Unfavorable attitude	245			38.9		

### Factors associated with knowledge regarding cervical cancer screening

For *P* < 0.25, age, marital status, ethnicity, education status, income, occupation, history of MSP, history of STD, use of modern FP method, and history of smoking were the covariates of knowledge on cervical cancer screening that were statistically significant (P-values < 0.25).

Again, in multivariable logistic regression, the history of MSP [AOR: 1.768 (95% CI: 1.227, 2.549)], and modern FP method use [AOR: 2.238 (95% CI: 1.410, 3.554) were the significant (P-value ≤ 0.05) factors for cervical cancer screening knowledge (Table 4). Factors related to the attitude towards cervical cancer screening. Similarly, for binary logistic regression, income, marital status (single, divorced, or widowed), education status, age at first sex, use of modern FP method, duration of modern FP uses more than five number of pregnancies, and history of STDs were the confounders for attitude towards cervical cancer screening (P-values < 0.25).

**Table 4:** Bivariable and multivariable analysis of factors associated with knowledge of cervical cancer screening among women in Debre Markos town, Northwest ethiopia, 2018.

Variable	knowledge		Crude OR [95%CI]	AOR [95%CI]
	Good knowledge	Poor knowledge		
Age of mothers				
40–49	65	56	1.331(0.893,1.984)	
30–39	309	200	1	
Marital status				
Others^**^	64	62	**1. 55(1.05**,**2.29)**	0.746 (0.487,1.141)
Married	310	194	**1**	1
Religion				
Muslim	19	10	0.76 (0.35,1.66)	
Christian and protestant	355	246	1	
Educational status				
Primary education	103	46	0.68(0.427,1.07)	
Secondary education	91	77	1.28(0.84,1.97)	
College and above	74	66	1.35(0.821,2.03)	
No formal education	106	70	1	
Ethnicity				
Others^***^	9	2	0.319(0.068,1.49)	
Amhara	365	254	1	
Occupation				
Self-employee	122	101	1.36(0.864,2.15)	
Private employee	98	44	0.74(0.442,1.234)	
Government employee	80	66	1.357(0.828,3.430)	
Housewife	74	45	1	
Household income ^****^				
900–1600	62	67	1.24(0.762,2.018)	
1601–2699	144	95	0.757(0.492,1.164)	
>=2700	98	33	0.386(0.245,2.22)	
<900	70	61	1	
Age started sexual intercourse for the first time.				
<=16	77	61	1.207(0.824,1.767)	
>16	297	195	1	
Multiple sexual partners				
Yes	178	76	**2.15 (1.54**,**3.011)**	**1.768 (1.23**, 2.55)^*^
No	196	180	**1**	**1**
History of smoking				
Yes	9	4	**3.37(1.03**,** 11.07)**	3.057 (0.896, 10.429)
No	247	370	**1**	1
History of STD				
Yes	48	40	**1.926(1.22**,**3.034)**	1.446 **(**0.875, 2.389)^*^
No	208	334	**1**	1
Ever use a modern FP method				
Yes	328	200	1.997(1.30,3.06)	**2.238(1.41**,** 3.55)**
No	46	56	**1**	**1**
Duration of modern FP method usage				
>3years	65	37	1.08 (0.691,1.695)	
1–3 years	263	162	1	
Family history of cervical cancer				
Yes	45	25	1.26(1.54,2.12)	
No	329	231	1	
Ever gotten pregnant.				
Yes No	319 55	206 50	1.41 (0.924,2.145) 1	
Gravidity				
>5 1–5	247 72	161 45	0.959(0.629, 1.46) 1	
Ever given birth				
Yes No	309 65	197 59	1.42 (0.959, 2.114) 1	
Parity				
>5	56	33	1.096(0.683,1.758)	
1–5	254	164	1	

** single, divorced and widowed, ***Oromo and Gurage, **** in Ethiopian Birr.

With controlling for the effect of other variables in multivariable logistic regression analysis, an education level (College and above education level [AOR: 2.006, 95%CI: 1.147, 3.508)]) marital status [AOR: 2.101 (95% CI: 1.219, 3.620)], and gravidity [AOR: 1.830 (95% CI: 1.125, 2.976)], were the significant factors (P-value ≤ 0.05)of attitude towards cervical cancer screening (Table 5).

**Table 5:** Bivariable and multivariable analysis of factors associated with attitude towards cervical cancer screening among women in Debre Markos town, Northwest ethiopia, 2018.

Variable	Attitude			Crude OR [95%CI]	AOR [95%CI]
	Favorable	Un favorable			
Marital status					
Married	323		181	**1**	**1**
Others^**^	62		64	**1.842(1.242**,**2.73)**	**2.101 (1.22**,**3.62)**
Educational status Primary education	99		50	0.862(0.546,1.363)	
Secondary education	100		68	1.161(0.752,1.793)	
College and above	75		52	1.412(0.896,2.225)	**2.01(1.15**,** 3.51)**
No formal education	111		65	1	**1**
Household income ^****^ 900–1600	72		57	1.62(0.979,2.68)	
1601–2699	141		89	1.422(0.910,2.223)	
>=2700	84		47	1.145(0.687,1.908)	
<900	88		43	1	
Age at first sex					
<=16	76		62	1.377(0.940,2.018)	
>16	309		183	1	
History of STD					
Yes	44		44	**1.697(1.08**,**2.668)**	1.28 (0.719, 2.282)
No	201		341	**1**	1
Ever use a modern FP method					
Yes	336		192	1.893(1.235,2.90)	1.244(0.709, 2.18)
No	49		53	**1**	1
Duration of modern FP method usage >3years	58		44	0.704 (0.454,1.093)	
1–3 years	277		148	1	
Gravidity					
>5	54		63	**1.659(1.09**,** 0.517)**	**1.830(1.125**,**2.98)**
1–5	139		269	**1**	**1**

** single, divorced and widowed, ***Oromo and Gurage, **** in Ethiopian Birr.

## Discussion

This study aimed to explore the knowledge and attitudes of women in Debre Markos town, Northwest Ethiopia, regarding cervical cancer screening. The findings revealed that 59.4% of participants were knowledgeable about cervical cancer screening, and 61.1% had a positive attitude towards it. The level of knowledge and attitude observed in this study appears to be lower than studies done in Yunnan province in China (69.8%)^24^, Kampala, Uganda (85.1%)^25^, and South Gondar (64%)^26^. Similarly, the attitude of cervical cancer screening done in Gondar, Northwest Ethiopia (67.7%)^27^ and (71.7%) in Adama, Oromia Region^28^, which are much higher than the findings of the present study. This difference could be due to several factors. One reason might be the age group of the participants, as different age categories may have varying levels of exposure to information. The time the studies were conducted could also play a role, especially if awareness-raising activities or media coverage were more active during that period. Moreover, educational background is an important factor; those with better access to formal education may be more likely to understand and respond positively to health-related issues. Therefore, differences in socio-demographic characteristics might partly explain the lower results in our study.

However, these study findings on cervical cancer screening knowledge were higher than those from studies in Central Nepal (46.5%)^29^, Shinyanga Region in Tanzania (8.1%)^30^, Diredawa city, Eastern Ethiopia (9.3%)^31^, Northwest Ethiopia (43.8)^32^, and Adigrat town, Northwest, Ethiopia (46.4%)^33^. Similarly, this study’s attitude towards cervical cancer screening was higher than studies done in central Nepal (51.8%)^29^, Adigrat town, Northwest, Ethiopia (53.3%)^33^, and Northwest Ethiopia (30.7%)^32^. This difference might be explained by variations in the study populations, where they live, and whether screening services are easily available. In areas where health care services are limited or hard to reach, women are less likely to have the information or motivation needed to seek screening.

Women’s knowledge and attitude towards cervical cancer screening were influenced by several interrelated factors. Women who had a history of MSPs were more likely to be knowledgeable about cervical cancer screening. Women with a history of MSPs were 1.768 times more likely to be knowledgeable about cervical cancer screening than those without MSPs [AOR: 1.768 (95% CI: 1.227, 2.549)], which was also supported by studies in Khartoum, Sudan^34^, and Lima City, Peru^35^. This may be because women with MSPs are more likely to visit health facilities for STDs, which increases their exposure to information about screening.

Similarly, women who had used modern family planning methods were also more knowledgeable about cervical cancer screening. Those who had ever used modern contraceptives were 2.238 times more likely to be knowledgeable about the screening [AOR: 2.238 (95% CI: 1.410, 3.554)]. This finding is supported by studies in Sadi Chanka district, West Ethiopia^36^ and Kabarole, Uganda^37^, suggesting that family planning services may provide women with opportunities to receive education on cervical cancer.

This study also found that women who were single, divorced, or widowed were significantly more likely to have a favorable attitude towards cervical cancer screening compared to married women [AOR: 2.101; 95% CI: 1.219, 3.620]. This finding is in line with a study conducted in Nairobi, Kenya, which also reported higher screening-related attitudes among unmarried women^38^. One possible explanation for this could be that single or divorced women may perceive themselves to be at greater risk, possibly due to having MSPs, and, as a result, may be more proactive in seeking preventive health services such as cervical cancer screening. Additionally, unmarried women might experience fewer social or familial constraints when making decisions related to their health, thereby enabling more positive health-seeking behavior.

Educational level was also a significant factor in shaping attitudes towards cervical cancer screening. Women with higher education level were 2.01 times more likely to have a positive attitude towards cervical cancer screening compared to those with no formal education [AOR: 2.01 (95% CI: 1.147, 3.508)]. This is supported by the findings from studies in Debre Tabor Town, Ethiopia^39^, and Anuppur District, India^40^, suggesting that education plays an important role in increasing awareness and shaping attitudes.

Finally, the number of pregnancies was another factor influencing attitudes towards cervical cancer screening. Women who had more than five pregnancies were 1.830 times more likely to have a positive attitude towards screening compared to those who had fewer pregnancies [AOR: 1.830 (95% CI: 1.125, 2.976)]. The finding is also supported by studies in Lima City, Peru^35^, Gondar Town^41^ and Arbaminch Town^42^. A possible explanation is that women with higher parity tend to have more frequent contact with health facilities, whether through antenatal care, delivery services, or follow-up visits, which increases their exposure to health education messages, including those related to cervical cancer screening. These repeated interactions with healthcare providers may help improve their awareness and positively shape their attitudes toward preventive services.

This study was conducted in an urban setting with relatively better access to health information and services, which may limit the generalizability of the findings to rural or underserved regions of Ethiopia. Given the country’s wide regional diversity in education, healthcare access, and cultural norms, the results may not fully reflect the national context.

## Conclusion

This study demonstrated that women in Debre Markos town had low levels of knowledge and unfavorable attitudes towards cervical cancer screening. Knowledge was significantly influenced using modern family planning methods and a history of multiple sexual partners, while attitudes were shaped by education level, marital status, and gravidity.

We recommend that health authorities and care providers intensify community-based education focusing on the risks related to multiple sexual partners, the benefits of family planning, and the importance of education.

## Data Availability

The datasets used and/or analysed during the current study are available from the corresponding author on reasonable request.
